# Population-based survival for cancer patients in Saudi Arabia for the years 2005–2009

**DOI:** 10.1038/s41598-021-04374-6

**Published:** 2022-01-07

**Authors:** Mohamed Aseafan, Edward Devol, Mahmoud AlAhwal, Riad Souissi, Reham Sindi, Haya AlEid, Shouki Bazarbashi

**Affiliations:** 1 Section of Medical Oncology, Oncology Center, King Faisal Specialist Hospital and Research Center, Riyadh, 11211 Saudi Arabia; 2 Department of Biostatistics, Epidemiology and Scientific Computing, King Faisal Specialist Hospital and Research Center, Riyadh, 11211 Saudi Arabia; 3 Department of Medicine, Faculty of Medicine, King Abdulaziz University, Jeddah, Saudi Arabia; 4 Research Department, Elm Company, Riyadh, Saudi Arabia; 5 Department of Therapeutic Services, Ministry of Health, Riyadh, Saudi Arabia

**Keywords:** Cancer epidemiology, Epidemiology

## Abstract

The Saudi Cancer Registry reported in 2007 the 5-year observed survival for the most common cancer sites for the years 1994–2004. In this report we looked at the cancer survival in the period 2005–2009 and evaluated the trend over the 15 years period from 1994 to 2009. Cases of the top 14 cancer sites reported by the population based Saudi Cancer Registry from 1 January 2005 to December 31, 2009, were submitted for survival analysis. The vital status of those patients was collected. Analysis of survival for the above period was compared with the prior reported 2 periods (1994–1999, 2000–2004). In addition, analysis was done according to age, sex, disease stage and the province. Data of 25,969 patients of the commonest cancer sites were submitted. Of those 14,146 patients (54%) had complete demographic data available and vital status was reported. Thyroid cancer had the highest 5- year observed survival of 94% (95% confidence interval (CI) 93–95%)), followed by Breast (72%, 95% CI 71–74%). In hematological malignancies, Hodgkin’s Lymphoma had the highest 5-year survival of 86% (95% CI 84–88%). Survival rates has improved in most of the cancers sites for the studied periods except for lung, uterine and Hodgkin’s lymphoma which plateaued. Our study confirms a steady improvement in the 5-year observed survival over time for the majority of cancers. Our survival data were comparable to western countries. This data should be used by policy makers to improve on cancer care in the kingdom.

## Introduction

National cancer statistics represent important data on which decision makers depend for national cancer control strategies. The most frequently reported statistic is the cancer incidence, reported as the age standardized rate per 100,000. Less commonly, data on mortality and survival are reported. Changes in cancer incidence in a country might reflect the efficacy of the measures for primary cancer prevention adopted in that country. On the other hand, survival statistics likely represent the efficacy of the preventive measures and therapeutic measures done for cancer in any population. Contrary to other chronic illnesses, survival in cancer patients represents one of the most significant outcome measures to be followed, despite the fact of its complexity being related the stage at diagnosis, the treatment offered, and other factors related to the health care system in the country.

The Kingdom of Saudi Arabia has an established national cancer registry since 1994 which reports mainly cancer incidence of the different cancer types and in the different regions of the Kingdom. Several attempts have been made to collect mortality data but were not successful. Despite an initial increase in cancer incidence, the Saudi cancer age standardized rate has plateaued and remains one of the lowest in the region. In 2007 the Saudi cancer registry reported the 5-year observed survival of the most common cancers for the years 1994–2004. The data represented the first national survival data reported across the region.

The health care system in Saudi Arabia is rapidly evolving with new medical cities and comprehensive cancer centers being established in most of the regions of the country over the past 10 years. In this report we have looked at the changing trend in survival for the most common cancers in Saudi Arabia between 1994 and 2009. Survival was calculated at 5-year intervals to establish the presence of change in recent years. In addition, we have looked at recent survival in different regions of the country to correlate with the availability of a comprehensive cancer care in each region.

## Methods

Saudi citizens diagnosed with cancer are registered by the Saudi Cancer Registry (SCR). Cancer registration is an active process. Through the Cancer Registry offices located in the various provinces within the country, trained registrars are dispatched to every hospital, clinic or laboratory and register every newly diagnosed cancer patient. Large cancer centers with hospital-based registries transfer their data electronically to the main office of the Saudi Cancer Registry. The Saudi cancer registry collects demographic data including full name, national identification number, date of birth, region where diagnosis was made and topography of the cancer. First line management and vital status at follow up are collected only if time permits and accordingly are not complete. Cancers are defined according to the International Classification of Disease-Oncology. The SCR uses the CanReg-4 program provided by the International Agency for Research on Cancer (IARC, Lyon, France). Cancer incidence data are reported annually, and reports are published at the Saudi Health Council website. In addition, SCR data are reported in the Cancer in the Five Continents series with the latest being volume X^1^. In order to know the vital status of each patient diagnosed, a communication was done with Elm Company, a digital transformation company that delivers an integrated business solution for both public and private sectors in many different fields, including various data services for individuals and vehicles thru its partnership with the National Information Center.

Elm provided data related to cancer patients, including the date of death of those deceased. An abstract of all registered (01 January 2005 through 31 December 2009) cases from a list of the most common cancers (top 14 cancer sites) was requested from the SCR database. The abstract included each case’s name, national identification number, date of birth and type of cancer. The vital status of those patients diagnosed was collected on 28 August 2019 and this date was considered the censoring date for those who were alive. In order for Elm company to report the vital status on the SCR abstract given to them, the patient’s name (full name in Arabic which includes given name, father’s name, grandfather’s name, and family name), national identification number and date of birth had to match. Those with un-matching data were not reported to us.

The study was approved by the institutional review board at King Abdulaziz University Hospital. Since these were national data, we were exempted from obtaining informed consent from patients by the Ethics Committee at King Abdulaziz University Hospital. Patient data were treated with strict confidentiality. Data were analyzed anonymously.

Survival was calculated from date of diagnosis to date of death or date of data capture (censoring) by the National Information Center. The Kaplan–Meier plot was used to calculate the survival according to age at diagnosis (≤ 40 years and > 40 years), sex, the province where the cancer diagnosis was made and disease stage. JMP statistical program (JMP version 15.0, 100 SAS Campus Drive, Cary, NC 27,513 USA) was used for the Kaplan–Meier plots. Since the data for the period 1994–2004 were already published, the above analysis was made only for the later period of 2005–2009.

## Results

A total of 31,285 patients diagnosed with the studied cancer sites were registered by the SCR between 1 January 2005 and 31 December 2009. Of those, 5,316 were excluded initially due to unavailability of complete national ID. Accordingly, 25,969 patients were submitted to Elm for vital status verification. The number of patients submitted per cancer site is illustrated in Table 1. A total of 14,146 patients (54%) of the submitted (45.2% of the total diagnosed with the studied cancer sites for the above period) were analyzed after Elm reported back to us on their vital status. We excluded patients with illogical date of diagnosis in relation to date of death in 228 patients (0.9%), and patients with secondary malignancy in 148 patients (0.6%). Reasons for not reporting the rest by Elm were (1) un-matching patient data with Elm records in 11,135 patients (43%), (2) missing other data (such as date of birth) in 106 patients (0.4%), and (3) duplicate national ID in 206 patients (0.8%). Breakdown of the number submitted and analyzed per age, sex and stage is also illustrated in Table 1. It is apparent that the analyzed cases reflect a similar demographic distribution to the submitted cohort. The exception was thyroid cancer where 45.8% of the submitted cohort were more than 40 years while it was 86.2% in the analyzed cohort. Similarly, males represented 21.3% of the submitted cohort vs 37.9% of the analyzed one. The analysis below is based on the 14,146 patient cases returned.

**Table 1:** Number and percentage of patients submitted versus analyzed according to age, sex, and cancer stage.

Cancer site	Submitted patients									Analyzed patients								
	Total Submitted	Age > 40 (%)	Age ≤ 40 (%)	Male (%)	Female (%)	Localized (%)	Regional (%)	Metastatic (%)	Unknown (%)	Total Analyzed	Age > 40 (%)	Age ≤ 40 (%)	Male (%)	Female (%)	Localized (%)	Regional (%)	Metastatic (%)	Unknown (%)
Stomach	1092	87.7	12.3	62.5	37.5	20.3	36.1	31.1	12.5	509	83.3	16.7	65	35	14.3	37.6	38.1	10
Colo-rectal	3638	85.4	14.6	55.6	44.4	21.9	41.1	29.4	7.6	1891	85.5	14.5	56.5	43.5	14.6	35.8	43.1	6.5
Liver	1599	93.6	6.4	71.3	28.7	30	11.2	16.1	42.7	731	94.8	5.2	72.6	27.4	34.6	13.3	17.9	34.2
Lung	1485	94.3	5.7	76.7	23.3	9.4	14.2	57.9	18.5	767	93.9	6.1	79.8	20.2	6.5	17.3	61.7	14.5
Skin	1024	84.6	15.4	59.7	40.3	68.2	8.5	3.4	19.9	427	93.4	6.6	65.4	34.6	59.3	13.2	6.6	20.9
Breast	4780	71.2	28.8	0	100	30.5	46.2	16.6	6.7	2762	70.3	29.7	0	100	17.3	45.5	31.4	5.8
Uterine	734	92.5	7.5	–	100	50.4	25.3	15	9.3	412	97.2	2.8	–	100	37.4	28.5	27.9	6.2
Ovarian	658	71.4	28.6	–	100	18.8	12.9	58.4	9.9	383	81.3	18.7	–	100	4.7	12.2	78	5.1
Prostate	1001	98.9	1.1	100	–	44.5	8.4	32.6	14.5	463	98.5	1.5	100	-	34.7	10.1	41.2	14
Bladder	1138	91.3	8.7	83	17	60	16.6	12.2	11.2	532	94.9	5.1	84.9	15.1	52.9	22.1	15.4	9.6
Thyroid	2409	45.8	54.2	21.3	78.7	50.7	32.2	7.3	9.8	1508	86.2	13.8	37.9	62.1	26.4	35.1	28.7	9.8
HL^1^	1355	27.7	72.3	58.4	41.6	14.7	29.2	42.3	13.8	896	38.9	61.1	59.4	40.6	8.6	24	54.9	12.5
NHL^2^	2661	67.2	32.8	60.4	39.6	19.6	17.6	45.4	17.4	1523	76.5	23.5	65.4	34.6	15.9	14.4	53.9	15.8
Leukemia	2395	42.9	57.1	58.1	41.9	–	–	–	–	1504	37.7	62.3	64.9	35.1	–	–	–	–

^1^HL: Hodgkin Lymphoma, ^2^NHL: Non-Hodgkin Lymphoma.

**Figure 1:**
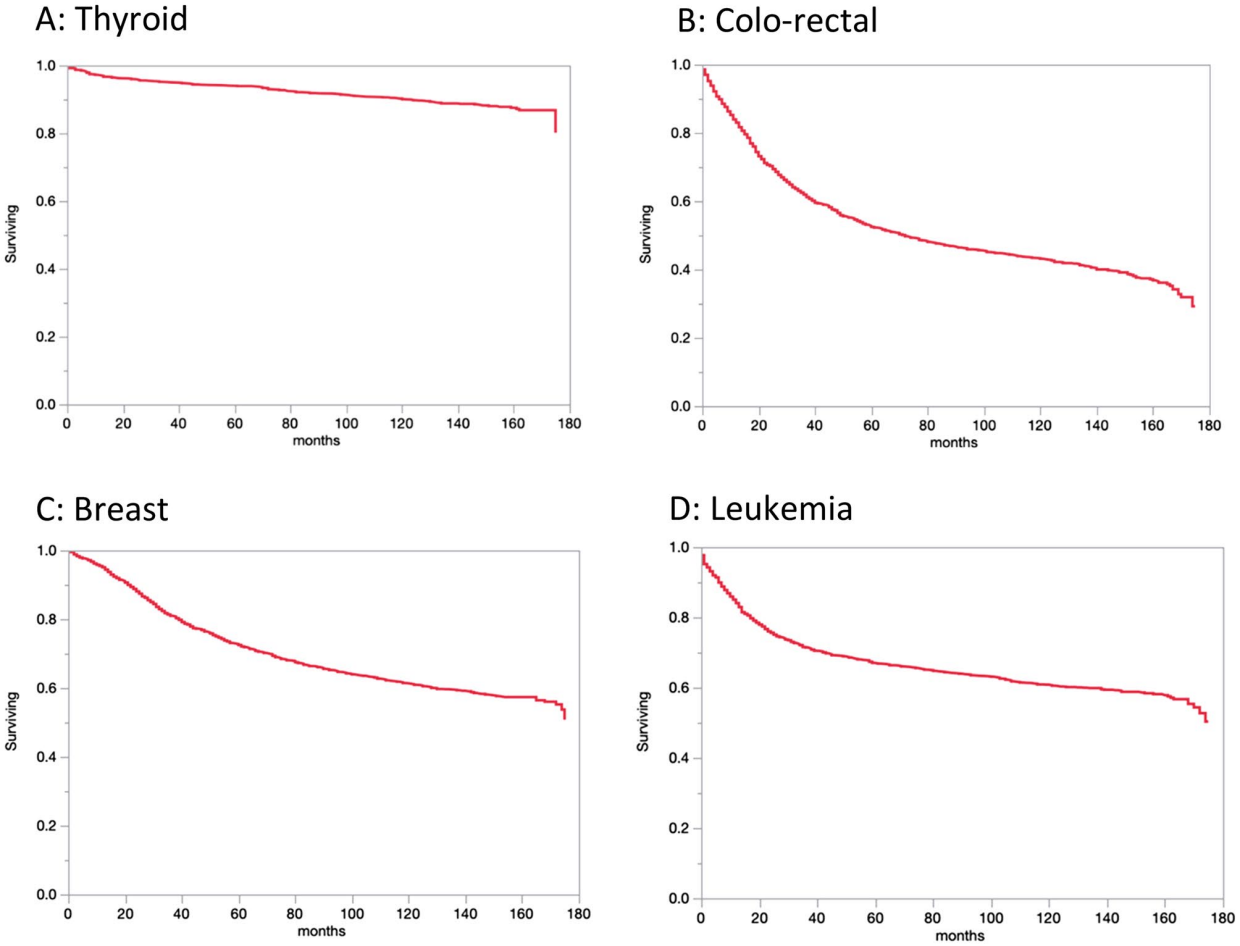
Kaplan Meier blot for observed survival for selected cancer site: (**A**) Thyroid, (**B**) Colo-rectal, (**C**) Breast, (**D**) Leukemia.

5-year observed survival for the different cancers diagnosed in the period 2005–2009 is provided in Table 2. The Kaplan Meier curves of selected common cancer sites is represented in Fig. 1. Of note, the highest survival in solid tumors was observed in thyroid cancer with a 5-year observed survival of 94% (95% confidence interval (CI) 93–95%), followed by breast (72%,95% CI 71–74%), followed by uterine cancer (68%, 95% CI 63–72%), followed by bladder cancer (59%, 95% CI 55–64%) followed by ovarian cancer (54%, 95% CI 49–59%). In hematological malignancies, Hodgkin’s Lymphoma had the highest 5-year survival (86%, 95% CI 84–88%), followed by leukemia (acute and chronic, 67%, 95% CI 64–69%) followed by non-Hodgkin’s lymphoma (64%, 95% CI 62–66%).

**Table 2:** 5-year observed survival for different cancers diagnosed in the period 2005–2009.

Cancer site	2005–2009 (95% CI)^1^
Stomach	24% (20%, 28%)
Colo-rectal	52% (50%, 55%)
Liver	19% (16%, 22%)
Lung	11% (9%, 13%)
Breast	72% (71%, 74%)
Uterine	68% (63%, 72%)
Ovarian	54% (49%, 59%)
Prostate	49% (45%, 54%)
Bladder	59% (55%, 64%)
Thyroid	94% (93%, 95%)
HL^2^	86% (84%, 88%)
NHL^3^	64% (62%, 66%)
Leukemia	67% (64%, 69%)

^1^CI: confidence interval, ^2^HL: Hodgkin Lymphoma, ^3^NHL: Non-Hodgkin Lymphoma.

The 5-year observed survival for different cancer sites in relation to age (≤ 40 years versus > 40 years) is illustrated in Table 3. Of note, patients ≤ 40 years of age did better in almost all cancer sites except for breast and prostate cancer and both results were not statistically significant.

**Table 3:** 5-year observed survival for different cancer site for the year 2005–2009 according to age.

Cancer site	> 40 (95% CI^1^)	** ≤ **40 (95% CI)	*P* value
Stomach	23% (21%, 25%)	26% (17%, 35%)	0.3110
Colo-rectal	52% (50%, 54%)	54% (51%, 57%)	0.0145
Liver	16% (14%, 18%)	49% (37%, 61%)	< .0001
Lung	11% (9%, 13%)	20% (9%, 31%)	0.0269
Skin	73% (69%, 77%)	92% (89%, 95%)	< .0001
Breast	73% (71%, 75%)	72% (69%, 75%)	0.8188
Uterine	66% (62%, 70%)	90% (85%, 95%)	0.0004
Ovarian	43% (37%, 49%)	75% (71%, 79%)	< .0001
Prostate	49% (45%, 53%)	28% (0%, 62%)	0.6278
Bladder	57% (53%, 61%)	77% (67%, 87%)	< .0001
Thyroid	87% (85%, 89%)	99% (99%, 100%)	< .0001
HL^2^	66% (58%, 74%)	90% (89%, 91%)	< .0001
NHL^3^	54% (51%, 57%)	78% (75%, 81%)	< .0001
Leukemia	51% (46%, 56%)	71% (69%, 73%)	< .0001

^1^CI: confidence interval, ^2^HL: Hodgkin Lymphoma, ^3^NHL: Non-Hodgkin Lymphoma.

Table 4 illustrate the 5- year observed survival for the different cancer sites according to sex. Findings indicate that males had worse survival in the following primary cancer sites (stomach, liver, lung, skin, thyroid, non-Hodgkin lymphoma and leukaemia). However, colo-rectal, bladder cancers and Hodgkin’s Lymphoma did not have a significant difference between males and females.

**Table 4:** 5-year observed survival for different cancer site for the year 2005–2009 according to sex.

Cancer site	Male (95% CI^1^)	Female (95% CI)	*P* value
Stomach	16% (12%, 20%)	36% (30%, 42%)	< .0001
Colo-rectal	51% (48%, 54%)	54% (51%, 57%)	0.2344
Liver	16% (13%, 19%)	26% (20%, 32%)	< .0001
Lung	07% (5%, 9%)	26% (20%, 32%)	< .0001
Skin	72% (67%, 77%)	83% (77%, 89%)	0.0036
Bladder	60% (56%, 64%)	57% (47%, 67%)	0.7257
Thyroid	88% (85%, 91%)	95% (94%, 96%)	< .0001
HL^2^	86% (84%, 88%)	85% (82%, 88%)	0.9801
NHL^3^	62% (59%, 65%)	67% (63%, 71%)	0.0071
Leukemia	64% (61%, 67%)	71% (68%, 74%)	< .0001

^1^CI: confidence interval, ^2^HL: Hodgkin Lymphoma, ^3^NHL: Non-Hodgkin Lymphoma.

Survival according to stage of disease is shown in Table 5. As expected, the 5-year observed survival was higher for localized disease in all cancers followed by regional and metastatic disease.

**Table 5:** 5-year observed survival for different cancer site for the year 2005–2009 according to disease stage.

Cancer site	Localized (95% CI^1^)	Regional (95% CI)	Metastatic (95% CI)	Unknown (95% CI)	*P* value^4^
Stomach	39% (29%, 49%)	26% (20%, 32%)	12% (8%, 16%)	24% (12%, 36%)	< .0001
Colo-rectal	73% (69%, 77%)	65% (62%, 68%)	21% (18%, 24%)	43% (34%, 52%)	< .0001
Liver	29% (24%, 34%)	13% (6%, 20%)	09% (4%, 14%)	14% (10%, 18%)	< .0001
Lung	36% (24%, 48%)	17% (11%, 23%)	06% (6%, 6%)	11% (5%, 17%)	< .0001
Skin	81% (77%, 85%)	61% (45%, 77%)	30% (7%, 53%)	73% (63%, 83%)	< .0001
Breast	88% (86%, 90%)	76% (74%, 78%)	35% (31%, 39%)	65% (58%, 72%)	< .0001
Uterine	81% (76%, 86%)	63% (54%, 72%)	28% (17%, 39%)	71% (55%, 87%)	< .0001
Ovarian	90% (83%, 97%)	67% (55%, 79%)	38% (32%, 44%)	62% (43%, 81%)	< .0001
Prostate	70% (64%, 76%)	43% (29%, 57%)	23% (16%, 30%)	42% (29%, 55%)	< .0001
Bladder	71% (66%, 76%)	41% (31%, 51%)	22% (11%, 33%)	59% (45%, 73%)	< .0001
Thyroid	98% (97%, 99%)	94% (92%, 96%)	58% (48%, 68%)	91% (86%, 96%)	< .0001
HL^2^	94% (90%, 98%)	90% (87%, 93%)	82% (78%, 86%)	78% (70%, 86%)	0.0007
NHL^3^	78% (74%, 82%)	75% (70%, 80%)	57% (54%, 60%)	50% (43%, 57%)	< .0001

^1^CI: confidence interval, ^2^HL: Hodgkin Lymphoma, ^3^NHL: Non-Hodgkin Lymphoma, ^4^Log-rank test.

Table 6 demonstrates the 5-year observed survival for each cancer site in relation to Saudi Arabia’s provinces. Significant Difference between provinces were noticed in breast cancer in the Northern Province as they had a lower 5-year observed survival by approximately 10% than other provinces in the Kingdom. Similarly, bladder cancer had lower 5 year observed survival in both the Western and Southern provinces compared to other provinces by > 25% relatively.

**Table 6:** 5-year observed survival for different cancer site for the year 2005–2009 according to province.

Cancer site	Eastern (95% CI^1^)	Central (95% CI)	Western (95% CI)	Northern (95% CI)	Southern (95% CI)	*P* value^4^
Stomach	24% (16%, 32%)	27% (20%, 34%)	22% (16%, 28%)	14% (0%, 29%)	23% (13%, 33%)	0.7590
Colo-rectal	58% (53%, 63%)	52% (48%, 56%)	50% (46%, 54%)	56% (45%, 67%)	47% (39%, 55%)	0.2117
Liver	15% (9%, 21%)	20% (16%, 24%)	19% (13%, 25%)	08% (0%, 24%)	23% (13%, 33%)	0.4995
Lung	11% (7%, 15%)	15% (9%, 21%)	08% (5%, 11%)	23% (17%, 29%)	04% (0%, 10%)	0.0581
Skin	76% (68%, 84%)	80% (63%, 87%)	74% (67%, 81%)	73% (53%, 93%)	73% (60%, 86%)	0.1729
Breast	72% (69%, 75%)	75% (73%, 77%)	71% (68%, 74%)	65% (57%, 73%)	71% (64%, 78%)	0.0305
Uterine	73% (64%, 82%)	73% (65%, 81%)	66% (58%, 74%)	63% (43%, 83%)	50% (36%, 64%)	0.0740
Ovarian	51% (39%, 63%)	59% (51%, 67%)	54% (45%, 63%)	45% (24%, 66%)	50% (35%, 65%)	0.6437
Prostate	56% (48%, 64%)	45% (37%, 53%)	44% (36%, 52%)	44% (11%, 77%)	56% (40%, 72%)	0.1085
Bladder	68% (60%, 76%)	63% (55%, 71%)	51% (44%, 58%)	72% (54%, 90%)	45% (29%, 61%)	0.0111
Thyroid	92% (89%, 95%)	95% (93%, 97%)	94% (92%, 96%)	90% (83%, 97%)	94% (86%, 100%)	0.6371
HL^2^	87% (83%, 91%)	88% (85%, 91%)	83% (79%, 87%)	83% (73%, 93%)	84% (76%, 92%)	0.0588
NHL^3^	72% (67%, 77%)	63% (59%, 67%)	60% (56%, 64%)	68% (58%, 78%)	62% (54%, 70%)	0.0024
Leukemia	62% (57%, 67%)	66% (62%, 70%)	71% (67%, 75%)	62% (52%, 72%)	70% (63%, 77%)	0.2185

^1^CI: confidence interval, ^2^HL: Hodgkin Lymphoma, ^3^NHL: Non-Hodgkin Lymphoma, ^4^Log-rank test.

The 5-year observed survival for different cancer sites was also compared to previously reported survival for the same cancers by the SCR. Table 7 shows the 5-year observed survival for the different time periods of 1994–1999, 2000–2004 and 2005–2009. Despite drop in survival for many cancers for the period 2000–2004, the 5-year observed survival was consistently better in all cancers in the period 2005–2009.

**Table 7:** 5-year observed survival for different cancers over time.

Cancer site	1994–1999	2000–2004	2005–2009
Stomach	22.8%	18.8%	24%
Colo-rectal	44.7%	44.3%	52%
Liver	14.5%	08.6%	19%
Lung	13.8%	6.5%	11%
Breast	66.4%	63.1%	72%
Uterine	66.7%	76.3%	68%
Ovarian	46.7%	47%	54%
Prostate	37%	41%	49%
Bladder	44.7%	43.4%	59%
Thyroid	91.2%	89%	94%
HL^1^	86.8%	83.5%	86%
NHL^2^	51.7%	58.9%	64%
Leukemia	59.4%	64%	67%

^1^HL: Hodgkin Lymphoma, ^2^NHL: Non-Hodgkin Lymphoma.

## Discussion

This is the first study evaluating population-based cancer survival in the Kingdom of Saudi Arabia. The study reports the 5-year observed survival for Saudi patients diagnosed with different cancers in the period from 2005 to 2009. It also examined the difference in the survival according to age, sex, disease stage, province, and the temporal trend over a 15-year period.

In general, most registries report 5-year age standardized relative survival. Unfortunately, the required data to calculate this was not available for Saudi Arabia; hence, we are reporting 5- year observed survival.

Thyroid cancer had the best survival rate among solid tumors, similar to most reported registries. Thyroid cancer represents the second most common cancer in females and the third in the population in Saudi Arabia in the years reported^2,3^. Breast cancer represents the commonest cancer in Saudi females and in the Saudi population in general. Despite a 5-year observed survival of 72%, this represents a slightly lower value than western countries, though most of them report relative 5-year survival^4,5^. Reasons behind a lower survival are likely related to a relatively high percentage of presentation in advanced stage (12.5%) and low rate of screening^6^. Screening for breast cancer has developed in several phases yet has not materialized into a national screening program^7–10^. Several opportunistic screening campaigns took place in several cities of Saudi Arabia mostly by non-governmental organizations^10^.

Uterine cancer is generally related to nulliparity^11^, which is uncommon is the Saudi population. 5-year observed survival is similar to some western countries, while appears inferior to others like USA and UK which reported cancer specific survival^4,12,13^. Urinary bladder cancer is seen mostly in males with 84.4% of diagnosed cases in our study of male sex. Despite 10.7% of the patients studied being in metastatic stage, which is around double what is seen in the USA^14^, the 5-year observed survival of 59% was similar to that reported in the Spanish population based cancer survival report^4^.

Colorectal cancer represents the commonest cancer in Saudi males and the second in the whole population^3^. The 5-year observed survival was remarkably similar to western data despite the latter representing cancer-specific survival. SEER data reported 54.8 and 57.7% 5-year cancer specific survival for colorectal cancer between 1990–1994 for males and females, respectively. This figure improved with a hazard ratio for improvement of 0.70 (95% confidence internal 0.68–0.72) for the years 2004–2009 in white Americans^15^. Evidently, a major factor affecting our survival figures in colorectal cancer is the late presentation in Saudi population with 30% of our patients presenting in metastatic stage. In fact, data on colorectal screening rates in a sample of the Saudi population showed 5.6% screening rate with less than 1% utilizing colonoscopy^16^.

Prostate cancer incidence is surprisingly low in Saudi Arabia with age-standardized rate (ASR) of 6.3/100,000 in 2016^2^. This in contrast to a much higher reported incidence in USA and UK with ASR of 109 and 170 per 100,000 respectively^17,18^. The reason for the low incidence may relate to it being secondary to the lack of PSA screening^19^. This might also be the reason for a higher incidence of advanced stage at presentation with 32% being metastatic at presentation and the lower 5-year observed survival of 49%.

Liver cancer typically develops in patients with liver cirrhosis. The prevalence of hepatitis B infection in Saudi patients diagnosed with hepatocellular carcinoma (HCC) was reported to be 67% (95% CI: 57.7–75.3), with a much lower incidence of 11.9 for Hepatitis C virus^20^. Screening plays an important role in the detection of early stage HCC, which might make it more amenable for curative surgery^21^. Despite the implementation of a national vaccination program for hepatitis B virus in 1989 in Saudi Arabia, the effect on HCC incidence has been modest^22^. Additionally, unfortunately no systematic screening for HCC is present in patients with cirrhosis, and hence the low 5-year observed survival rate of 19%.

The hematological malignancies in Saudi Arabia have encouraging survival rates compared to western data^4^. Reasons behind this could be a usual early diagnosis in hematological malignancies and the available tertiary care centers that accept such cases promptly.

The survival difference by age has public health implications that need attention. However, caution should be taken as our study reports observed survival rather than net survival which adjust for the competing causes of death which increase in older population. In our study, younger patients did better in terms of survival similar to other countries. The exception was breast and prostate cancer, and both were not statistically significant^4,15,23,24^. Only 5 patients were younger than 40 years of age with prostate cancer which is expected due to higher median age at diagnosis. Older patients tend to have lower survival that may be explained by other comorbidities competing for death and are less likely to get aggressive therapy^23^.

In our study, sex has displayed a prognostic role with females having better survival across all reported cancer sites except in urinary bladder cancer where the difference was not statistically significant. It is important to note that urinary bladder cancer occurs predominantly in males in our region^2,3^. The survival superiority for females in cancer has been previously described in many populations in the literature^4,23,25^. Many hypotheses were proposed, of which that sex hormone patterns in female could play a role in providing a superior survival in females compared to males^4^.

Cancer stage at diagnosis serves as one of the most important prognostic factors in generally all cancer sites. Patients with localized disease have an advantage in survival compared with locally advanced and metastatic disease in view of the possible surgical intervention. The staging system used in our study represents the one used by the CanReg-4 registry program supplied by IARC. We interpret our survival data with caution in view of the high number of unknown cancer stage in some sites. The lower survival seen in some cancers like stomach, lung and liver represent the usually advanced stage in which they present. In general, cancers which are known to have higher sensitivity to systemic therapies in advanced stages had markedly improved 5-year observed survival such as thyroid, Hodgkin’s and non-Hodgkin’s lymphoma.

Survival trends observed in our study confirmed the steady improvement in the 5-year observed survival over time. Lung and uterine cancer survival rates did plateau over the 15-year period studied. Hodgkin’s lymphoma had an excellent survival rate of 86% in the period 1994–1999 and this was maintained in the years 2005–2009. These survival trends need to be interpreted with caution as many factors might have played a role like health care accessibility, care quality, staff expertise, treatment availability and statistical artifacts^23^. One important factor is the continued improvement in anticancer therapy. Additionally, the health care system has advanced with time. This might be behind the improvement in the 5-year observed survival over time.

Survival results from the Northern province underperformed other provinces in the Kingdom of Saudi Arabia. This could be explained by lack of oncology centers and patient education in that province. According to the Saudi Ministry of Health achievements report 2013, the Northern province had six specialized medical center (tertiary hospitals) while Riyadh region has 22, and Makkah region has 36^26^.Acknowledging these defects helps to tackle these issues leading to improvement in survival.

Population-based survival may give an insight into the effectiveness of the health care system in managing cancer patients^23^. Achieving better cancer control is a global health challenge that is used as a tool to assess the health care system efficacy and helps as guide if improvement is required by comparing population survival rates with others. The CONCORD-3 study is one of the biggest global comparative studies with 322 separate registries in 71 countries covering approximately 1 billion people of the global population. It investigated patients diagnosed with cancer between 2000 and 2014 and has shown that survival trends were in general on an upwards trend. However, it was clear that high-income countries enjoy better survival than low-middle income countries^27^.

As the population in Saudi Arabia continues to grow, the incidence of cancer has increased from 1990 to 2016^28^. The SCR is the first national registry in Saudi Arabia established in 1992 by a resolution of the Minister of Health. It is one of first registries founded in the region. However, clearly efforts are needed to improve the registration process to get a more comprehensive survival and mortality data in the future.

Comparative survival results between populations and countries help health care officials to identify issues with the current health care system and possibly formulate national cancer strategies to improve cancer control^27,29^. The need for a national cancer screening programs in Saudi Arabia should be carefully studied. This is because the incidence of cancer in general in Saudi Arabia is much below western countries levels and the benefit of mass screening is not known.

Our study has several limitations. First, only 54% of cancer cases submitted were analyzed as the required data was not available for the others. An additional limitation was our inability to produce relative survival rates in view of the lack of the denominator data.

In summary, the relatively lower observed survival in some solid tumors in our study may represent the advanced stage at presentation. Hematological malignancies in Saudi Arabia have 5-year observed survival comparable to the developed countries. A positive trend in cancer survival over time was seen in most studied cancer sites. The Saudi cancer registry needs to develop a systematic method for capturing mortality and survival data with time. This study highlights the need for future studies assessing the role of screening programs for early diagnosis which could lead to improving the overall survival of cancer in the Kingdom of Saudi Arabia.

### Ethical approval

This study was performed in line with the principles of the Declaration of Helsinki. Approval was granted by the Ethics Committee at King Abdulaziz University Hospital (Date 21 April 2020/No. 215-20).

### Consent to participate

Since the study involve retrospective national registry data, we were exempted from obtaining informed consent from patients.

## Data Availability

All data and documents needed will be provided upon request through email: bazarbashi@kfshrc.edu.sa.
